# Quantitative Electroencephalogram Changes before and after Postoperative Delirium: A Prospective, Multicenter Cohort Study

**DOI:** 10.1097/ALN.0000000000006026

**Published:** 2026-03-09

**Authors:** Julia Van der A, Yorben Lodema, Lotus Ten Bosch, Anna B. Van Heesch, Simone J. T. Van Montfort, Ilse M. J. Kant, Claudia Spies, Edwin Van Dellen, Arjen J. C. Slooter

**Affiliations:** 1 Department of Intensive Care Medicine, University Medical Center Utrecht Brain Center, and Department of Psychiatry, University Medical Center Utrecht, Utrecht University, Utrecht, The Netherlands.; 2 Department of Psychiatry and University Medical Center Utrecht Brain Center, University Medical Center Utrecht, Utrecht University, Utrecht, The Netherlands.; 3 Department of Intensive Care Medicine, University Medical Center Utrecht Brain Center, and Department of Psychiatry, University Medical Center Utrecht, Utrecht University, Utrecht, The Netherlands.; 4 Department of Intensive Care Medicine, University Medical Center Utrecht Brain Center, and Department of Psychiatry, University Medical Center Utrecht, Utrecht University, Utrecht, The Netherlands.; 5 Department of Intensive Care Medicine, University Medical Center Utrecht Brain Center, University Medical Center Utrecht, Utrecht University, Utrecht, The Netherlands.; 6 Department of Intensive Care Medicine, University Medical Center Utrecht Brain Center, and Department of Radiology, University Medical Center Utrecht, Utrecht University, Utrecht, The Netherlands.; 7 Department of Anesthesiology and Operative Intensive Care Medicine (Campus Charité Mitte and Campus Virchow-Klinikum), Charité, Universitätsmedizin Berlin, Berlin, Germany.; 8 Department of Psychiatry, and University Medical Center Utrecht Brain Center, University Medical Center Utrecht, Utrecht University, Utrecht, The Netherlands; Department of Neurology, and Vrije Universiteit Brussel, UZ Brussel, Brussel, Belgium.; 9 Department of Intensive Care Medicine, University Medical Center Utrecht Brain Center, University Medical Center Utrecht, Utrecht University, Utrecht, The Netherlands; Department of Psychiatry, University Medical Center Groningen and University of Groningen, Groningen, The Netherlands.

## Abstract

**Background:**

Delirium occurs frequently after major surgery in older adults and is associated with long-term cognitive dysfunction. While postoperative delirium (POD) shows electroencephalographic (EEG) changes during the acute phase, it remains unclear whether quantitative EEG alterations precede or persist after POD. Identifying such patterns could reveal risk markers and mechanisms of long-term cognitive dysfunction.

**Methods:**

In this prospective, multicenter cohort study, EEG recordings were obtained in patients aged 65 yr or older before and 3 months after major elective surgery without preexistent cognitive dysfunction, and in nonsurgical controls. The authors analyzed quantitative EEG measures that show alterations during acute delirium, namely relative power, phase-based and amplitude-based functional connectivity, spectral variability, and signal complexity. Linear mixed models were used to assess effects of time, surgery, and POD on these EEG measures.

**Results:**

Of 379 enrolled surgical patients, 330 had sufficient EEG data quality, of whom 59 (18%) developed POD. Fifty-seven nonsurgical controls were included and served as a reference. At baseline, future POD patients exhibited significantly lower beta amplitude–based connectivity (β = –0.36; *P*_corrected_ = 0.04). No other EEG measures showed significant differences between groups at baseline. Three months after surgery, no persistent changes in quantitative EEG characteristics were observed in relation to POD occurrence or surgery alone.

**Conclusions:**

This study identified reduced preoperative beta amplitude–based connectivity as a possible marker of neurophysiological vulnerability for POD. However, the absence of significant longitudinal changes in quantitative EEG measures suggests that resting-state EEG networks may functionally recover or compensate in this cohort at 3 months postoperative.

## Abstract 2

Preexisting brain vulnerability is subtle. The preoperative electroencephalogram features in patients who went on to develop delirium were very similar to those in patients who did not develop postoperative delirium except for showing reduced functional connectivity in the beta frequency band. This reduction in connectivity was most marked in the central and parietal regions. For the whole patient group, there were no long-term alterations in electroencephalogram features at 3 months after surgery.

## Editor’s Perspective

**What We Already Know about This Topic**

- During episodes of delirium, the electroencephalogram (EEG) shows increased slow activity and diminished high-frequency activity and functional connectivity—signs of disruption of brain network integration.
- Previous work to capture the EEG characteristics of a brain that is vulnerable to subsequent delirium have shown conflicting results.
- It is also unclear whether the EEG changes seen during an episode of delirium, persist after the episode has apparently resolved.

**What This Article Tells Us That Is New**

- Preexisting brain vulnerability is subtle. The preoperative EEG features in patients who went on to develop delirium were very similar to those in patients who did not develop postoperative delirium except for showing reduced functional connectivity in the beta frequency band.
- This reduction in connectivity was most marked in the central and parietal regions.
- For the whole patient group, there were no long-term alterations in EEG features at 3 months after surgery.

Delirium is a neuropsychiatric syndrome characterized by acute disturbances in attention, awareness, and cognition, and is considered a clinical manifestation of acute encephalopathy.^1,2^ Postoperative delirium (POD) occurs in 15 to 25% of older individuals after major surgery and is associated with prolonged hospitalization, long-term cognitive dysfunction, and increased risk of dementia.^3,4^

A growing body of evidence suggests that from a neurobiological perspective, delirium may arise from the functional disruption or breakdown of neural networks, often described as a “disconnection syndrome.”^5–7^ Electroencephalography (EEG) provides a noninvasive method to study these network dynamics in delirium. EEG recordings during delirium have revealed characteristic patterns: a predominance of slow-frequency activity (delta and theta frequency bands), decreased power in faster frequency bands (alpha and beta frequency bands),^8–10^ reduced functional connectivity,^7,9,11^ changes in signal variability,^12^ and decreased signal entropy, indicative of altered complexity.^12–14^ These findings support the hypothesis that delirium involves disruption of brain network integration.

Although multiple studies have described quantitative EEG (qEEG) changes during delirium episodes,^9^ critical gaps remain in our understanding of EEG patterns related to delirium vulnerability and its long-term effects. Before delirium onset, nonmodifiable risk factors such as advanced age and cognitive impairment may lower baseline network connectivity,^5,6^ which can be further disrupted by precipitating factors including inflammation,^15^ ischemia,^16^ or anesthesia and surgery.^17,18^ Previous studies suggest that preoperative qEEG differences are present between patients who will and will not develop POD. Findings associated with increased POD risk include higher frontal connectivity,^15^ lower median dominant frequency,^19^ increased alpha and beta power,^20^ and reduced variations in occipital alpha power.^21^ However, interpretation of these findings is challenging as they appear contradictory, with reports suggesting potential compensatory mechanisms (*e.g.*, higher frontal connectivity) while others indicate neurophysiological vulnerability (*e.g.*, EEG slowing, lower dominant frequency). Prospective research measuring EEG before precipitating events is therefore essential to distinguish baseline neural vulnerability to delirium from effects of precipitating factors, which would increase understanding of delirium pathophysiology.

After delirium resolution, current evidence suggests that EEG changes may persist, but these studies were limited to dual-channel EEG recordings in a small nursing home population.^22,23^ Kim *et al*. observed significant postoperative decreases in median dominant frequency in both delirious and nondelirious patients.^19^ These changes in median dominant frequency persisted regardless of delirium status and highlight the need for a nonsurgical control group to differentiate between the long-term effects resulting from delirium and from anesthesia and surgery. Besides EEG slowing, more recent functional magnetic resonance imaging (fMRI) evidence revealed decreased connectivity strength and network alterations after delirium resolution.^24^ Understanding these long-term changes in brain activity could help explain why patients who have experienced delirium are at increased risk of dementia,^25^ as similar patterns of decreased functional connectivity, signal complexity, and EEG slowing have been observed in dementia.^26,27^

To examine preoperative qEEG differences and long-term qEEG changes associated with POD, we conducted a prospective, multicenter cohort study examining qEEG in surgical patients preoperatively and 3 months postoperatively. We compared three groups: patients who developed POD, those who did not, and nonsurgical controls. This design allowed us to distinguish EEG changes specifically associated with POD from those resulting from anesthesia and surgery. Our primary aims were to determine whether preoperative qEEG differences exist in patients who later develop POD, and whether the occurrence of POD, or undergoing anesthesia and surgery without the occurrence of POD, is associated with qEEG alterations that are evident 3 months postoperatively.

## Materials and Methods

### Study Design and Population

This study reports on a prespecified secondary endpoint of the Biomarker Development for Postoperative Cognitive Impairment in the Elderly (BioCog) project, conducted at the University Medical Center Utrecht (Utrecht, The Netherlands) and Charité Hospital (Berlin, Germany).^28^ Ethical approvals were obtained from both centers (approval Nos. 14-469 and EA2/092/14, respectively), and the trial was registered at ClinicalTrials.gov (NCT02265263) on September 23, 2014. Written informed consent was obtained from all participants before their inclusion in the study, and all procedures followed the latest version of the Declaration of Helsinki. Participants were included between October 2014 and December 2017. The study population consisted of individuals aged 65 yr or older of European ancestry who were scheduled for major elective surgery (*i.e.*, orthopedic, cardiac, gastrointestinal, gynecologic, urologic, maxillofacial, or otorhinolaryngologic surgery) of at least 60 min. Exclusion criteria included a life expectancy of less than 1 yr and signs of early dementia as indicated by a score of 23 or less on the Mini-Mental State Examination.^29^ Nonsurgical controls were community-dwelling individuals recruited *via* general practitioners in Utrecht and Berlin. Inclusion criteria were similar to those of the surgical group, except they had no scheduled major surgery. These nonsurgical controls provided a reference standard for normal qEEG stability during 3 months. None of these controls underwent surgery or experienced delirium during the study period.

### Clinical Assessment

Patients underwent a preoperative clinical assessment within 2 weeks before surgery. Preoperative physical status was scored using the American Society of Anesthesiologists (Schaumburg, Illinois) Physical Status classification.^30^ The Alcohol Use Disorders Identification Test assessed alcohol consumption, with a cutoff of 8 points indicating misuse.^31^ Depressive symptoms were evaluated using the 15-item Geriatric Depression Scale, with a score of 6 or greater defining depression.^32^ Functional impairment was measured using the Barthel Index.^33^ The continuous outcome measure was the total score (0 to 100), where the maximum score of 100 indicates fully independent functional ability. The Dutch reading test for adults “Nederlandse Leestest voor Volwassenen” was used to estimate premorbid intelligence quotient.^34^ Medical records were screened for hypertension, diabetes mellitus, and previous transient ischemic attack or stroke. If medical records were unavailable, patients were directly questioned about stroke history.

### Delirium Assessment

Delirium was defined according to the criteria outlined in the fifth edition of the *Diagnostic and Statistical Manual of Mental Disorders*.^2^ Trained research staff conducted delirium assessments once preoperatively and twice daily postoperatively until discharge, with a maximum follow-up of 7 postoperative days. This 7-day cutoff was established because delirium occurring beyond this point is unlikely to be directly related to anesthesia and/or surgery. Delirium assessments were performed using the Confusion Assessment Method for the Intensive Care Unit,^35^ the Nursing Delirium Screening Scale,^36^ and chart review.^37^ Patients were classified as delirious if they met any of the following criteria: a positive Confusion Assessment Method for the Intensive Care Unit score, 2 or more cumulative points on the Nursing Delirium Screening Scale, or a chart review indicating clear descriptions of delirium (*e.g.*, confused, agitated, drowsy, disoriented, delirious, or receiving antipsychotic therapy for delirium). In cases of uncertainty, a delirium expert (A.J.C.S.) was consulted for final determination. The duration of delirium was defined as the cumulative number of days a patient met these criteria.

### EEG Recordings, Selection, and Preprocessing

Five-minute resting-state EEG recordings were performed preoperatively (baseline) and 3 months postoperatively (or 3 months after baseline, in control participants) using a 32-electrode cap (Braincap MR, Brain Products GmbH, Germany) positioned according to the internationally standardized 10-20 electrode placement system. During the recordings, patients were awake and sitting upright with their eyes closed. The BrainVision Recorder (Brain Products GmbH) was used with a sampling frequency of 5,000 Hz, and electrode impedance was kept less than 5 kΩ.

Data preprocessing was conducted using MNE (version 1.6.1), an open-source package for EEG signal processing available for Python (Python Software Foundation, version 3.11, https://www.python.org/). Two trained researchers visually inspected EEG recordings for artifacts, including eye movements and blinks, muscle activity, cardiac signals, and electrode disturbances. Only artifact-free epochs were included. Channels with persistent artifacts and/or noise were interpolated. If more than three channels needed interpolation or if there were insufficient artifact-free epochs, the EEG recording was excluded from analysis. Channels TP9 and TP10 were excluded due to muscle artifacts, as well as the electrocardiography channel, leaving 29 channels available for analysis. The first eight artifact-free epochs of 8 s were selected for further analysis, which is sufficient for stable results.^38^ The data were re-referenced to an average reference and downsampled to 500 Hz. EEG data were then band pass–filtered using a zero-phase Hamming windowed-sinc finite impulse response (FIR) filter, with a transition bandwidth dynamically set to 10% of the cutoff frequency and constrained to a range of 0.4 to 1.5 Hz for all separated frequency bands: delta (0.5 to 4 Hz), theta (4 to 8 Hz), alpha (8 to 13 Hz), and lower beta (13 to 20 Hz). Beta band analysis was restricted to lower frequencies to minimize muscle artifact contamination.^39^

### qEEG Characteristics

qEEG measures were calculated using Python (version 3.11, MNE version 1.6.1, https://github.com/yorbenlodema/EEG-Pype). Figures were generated using the MNE-Python library (version 1.9.0) and Matplotlib (version 3.9.0, https://matplotlib.org). Metrics for analysis were selected based on previous literature indicating a potential association with delirium.^9,11–13^ Relative power was calculated by dividing the absolute power per frequency band (delta [0.5 to 4 Hz], theta [4 to 8 Hz], alpha [8 to 13 Hz] and beta [13 to 20 Hz]) by the total power. Peak frequency was defined as the frequency corresponding to the highest peak within the 4- to 13-Hz range. The mean phase lag index was calculated to quantify functional connectivity strength,^40^ measuring asymmetry in the distribution of instantaneous phase differences between signals, with values ranging from 0 to 1. The amplitude envelope correlation was used to measure functional connectivity by calculating a Pearson correlation between two amplitude envelopes. Time-domain orthogonalization was applied, resulting in the corrected amplitude envelope correlation (AECc).^41^ Functional connectivity was calculated per electrode pair (29 × 28 channels) per epoch and then averaged, which is in line with other applications of AECc and phase lag index in delirium research. Signal complexity in the time domain was quantified using approximate entropy (ApEn).^42^ ApEn quantifies the predictability of patterns in a time series by assessing their repeatability. ApEn was calculated with an embedding dimension of 2 and the tolerance parameter set to 25% of each individual channel’s SD, calculated for the entire broadband (0.5 to 20 Hz).^12^ Spectral variability was calculated using a 2-s sliding window with 50% overlap. SD was calculated for relative power across each consecutive window, reflecting variability of relative power in each frequency band across epochs.^12^ Based on previous findings of diffuse EEG alterations during delirium for a range of EEG characteristics, we calculated a global average for each measure using all 29 electrodes. Additionally, we computed averages for four regions of interest: frontal, central, parietal, and occipital. The specific electrodes comprising each region of interest are detailed in Supplemental Digital Content 1 (https://links.lww.com/ALN/E450).

### Statistical Analysis

We reported baseline characteristics (using medians and 25th to 75th percentiles) for the total sample and separately for three groups: surgical patients who developed POD (POD+), surgical patients who did not develop delirium (POD–), and nonsurgical controls. The following relationships were investigated: (1) whether baseline differences existed between POD+ and POD–, (2) whether the changes in qEEG measures from baseline to follow-up differed between POD+ and POD–, and (3) whether changes in qEEG measures differed between POD– and nonsurgical controls. To analyze these questions, we used linear mixed models (LMMs) for each qEEG characteristic with R (R version 4.1.1, package lmerTest, https://www.r-project.org, accessed April 2, 2026). Each model included the qEEG characteristic averaged across electrodes as the dependent variable, with fixed effects for center (0 = Utrecht, 1 = Berlin), age, sex (0 = female, 1 = male), timepoint (1 = preoperative/baseline, 2 = postoperative/follow-up), and group (0 = POD−, 1 = POD+, 2 = nonsurgical controls). Participant was included as a random effect (random intercept model), and the interaction term for timepoint by group was included to assess group-specific EEG changes over time. The full model specification was as follows:

qEEG = *b*_0_ + *b*_1_centre + *b*_2_age + *b*_3_sex + *b_4_*timepoint + *b_5_*group + *b_6_*(timepoint × group) + *u*_participant_ + *e*

In cases where a significant timepoint by group interaction was found for a global qEEG measure, follow-up exploratory analyses were planned by running the same LMM for each of the four regional (frontal, central, parietal, occipital) averages of that measure to identify the potential source of the effect. *Post hoc* standardization was performed using the “effectsize” package in R to convert raw model coefficients to standardized β coefficients (in SD units), facilitating comparison across qEEG measures. Standardized β coefficients were interpreted using Cohen’s conventions (small |β| ≈ 0.1, medium ≈ 0.3, large ≈ 0.5) as approximate effect size guidelines.^43^ All analyses testing statistical significance were two-sided, which was set at *P* ≤ 0.05. To account for multiple comparisons within a measure (*i.e.*, testing qEEG measures across the four frequency bands), we applied a false discovery rate (FDR) correction using the Benjamini–Hochberg procedure.^44^ However, given the study’s exploratory nature, no further corrections were applied for the number of global qEEG measures tested. As an additional exploratory spatial analysis, electrode-level LMMs were run for all qEEG measures, using the same model structure. False discovery rate correction was applied across all 29 electrodes within each qEEG measure separately.

## Results

### Study Population Characteristics

**Fig. 1.**
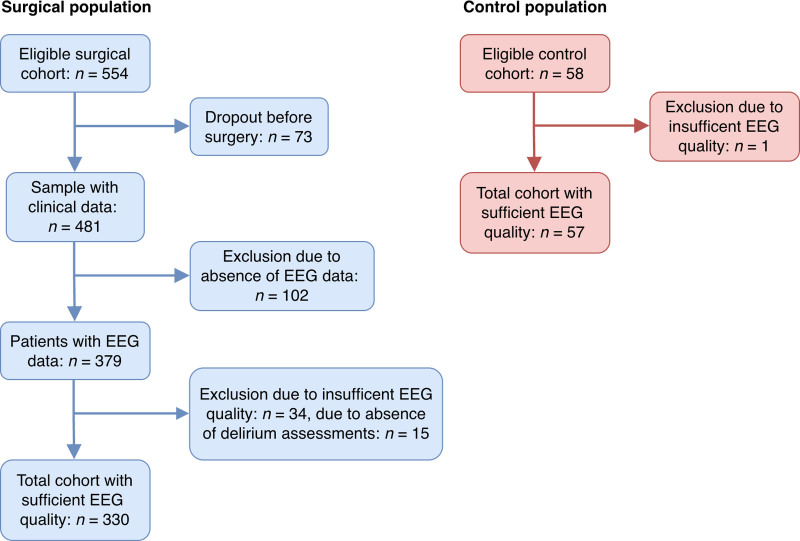
Flowchart of the inclusion of subjects. EEG, electroencephalography.

The eligible cohort consisted of 554 surgical patients and 58 nonsurgical controls. Of the surgical patients, 73 dropped out before surgery (*e.g.*, surgery was canceled, patients did not want to continue in the study), leaving 481 patients with clinical data. Subsequently, 151 patients were excluded (102 due to absent EEG data, 34 due to insufficient EEG quality, and 15 due to missing delirium assessments), resulting in 330 patients for final analysis (fig. 1). In the nonsurgical control group, 1 participant was excluded due to insufficient EEG quality, leaving 57 controls. A comparison of participants with complete *versus* incomplete or excluded EEG data (Supplemental Digital Content 2, https://links.lww.com/ALN/E450) showed similar baseline demographics, cognitive scores, and functional status across all groups, although complete cases were more likely to be recruited from the Utrecht center. Among surgical patients, 59 (18%) developed delirium within the first 7 postoperative days, with a median duration of 2 days. The POD+ group was slightly older, had more comorbidity at baseline, had longer anesthesia duration, and had extended hospital stays compared to the POD– group (table 1).

**Table 1.** Baseline Demographics and Clinical Characteristics

	Total Surgical Cohort (n = 330)	POD+ (n = 59)	POD– (n = 271)	Nonsurgical Controls (n = 57)
Characteristics at baseline				
Center Utrecht, No. (%)	160 (49%)	25 (42%)	135 (50%)	47 (82%)
Female, No. (%)	123 (37%)	27 (46%)	96 (35%)	27 (47%)
Age, yr	72 [68–75]	74 [72–76]	71 [68–75]	70 [67–75]
Diabetes mellitus, No. (%)	60 (18%)	19 (32%)	41 (15%)	10 (18%)
Body mass index	27 [24–29]	27 [23–29]	27 [24–29]	25 [24–28]
Transient ischemic attack or stroke, No. (%)	31 (9%)	10 (17%)	21 (8%)	5 (9%)
Charlson comorbidity index	1 [0–2]	1 [0–2]	1 [0–2]	—
Barthel Index	100 [100–100]	100 [98-100]	100 [100–100]	100 [100–100]
Mini-Mental State Examination	29 [28–30]	28 [27–29]	29 [28–30]	29 [28–29]
Premorbid IQ^*^	105 [97–113]	102 [94–110]	107 [98–114]	111 [98–115]
Alcohol misuse, No. (%)	20 (6%)	4 (7%)	16 (6%)	0 (0%)
Geriatric Depression Scale	1 [0–2]	1 [0–2]	1 [0–2]	1 [0–2]
ASA Physical Status, No. (%)				
I	21 (6%)	1 (2%)	20 (7%)	13 (23%)
II	204 (62%)	30 (51%)	174 (64%)	22 (61%)
III	105 (32%)	28 (47%)	77 (28%)	7 (16%)
Surgery characteristics				
Surgical specialty, No. (%)				
Cardiothoracic	49 (15%)	14 (24%)	35 (13%)	—
Intra-abdominal	103 (31%)	20 (34%)	83 (31%)	—
Orthopedic	102 (31%)	16 (27%)	86 (32%)	—
Other	76 (23%)	9 (15%)	67 (25%)	—
Duration of anesthesia, min	200 [109–288]	246 [168–339]	183 [101–265]	—
Length of hospital stay, days	5 [3–9]	10 [6–16]	4 [3–7]	—
Length of ICU stay, days	0 [0–0]	0 [0–1]	0 [0-0]	—
Delirium duration, days	—	2 [1-3]	—	—
Characteristics at follow-up				
Mortality before follow-up, No. (%)	8 (2%)	2 (3%)	6 (2%)	0 (0%)
Barthel Index	100 [96–100]	100 [95–100]	100 [100–100]	100 [100–100]

Values are median [25th–75th percentile] unless stated otherwise.

* Estimated using the Dutch version of the National Adult Reading Test.

ASA, American Society of Anesthesiologists; ICU, intensive care unit; IQ, intelligence quotient; POD+, surgical patients who developed postoperative delirium; POD–, surgical patients who did not develop postoperative delirium.

### qEEG Characteristics at Baseline

**Fig. 2.**
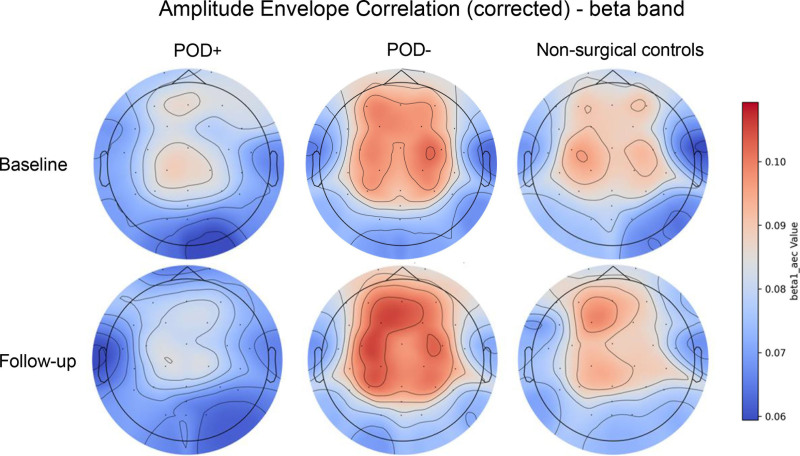
Corrected amplitude envelope correlation (AECc) of the beta (13 to 20 Hz) frequency band. The heat maps display the median beta AECc across three groups: surgical patients with postoperative delirium (POD+), surgical patients without POD (POD–), and nonsurgical controls at two different timepoints (baseline/preoperative and follow-up/3 months postoperative). At baseline, beta AECc was significantly lower in POD+ patients compared to POD–, while no significant main or interaction effects were observed from baseline to follow-up.

**Fig. 3.**
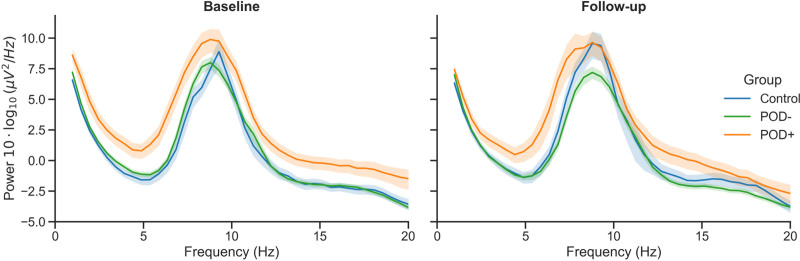
Power spectral density by group and timepoint. Group-averaged power spectral density of resting-state electroencephalography at baseline (preoperative, *left*) and follow-up (postoperative, *right*). *Blue* represents the nonsurgical control group (n = 57), *green* the surgical patients who did not develop postoperative delirium (POD–; n = 217), and *orange* the surgical patients who did develop postoperative delirium (POD+; n = 59). *Solid lines* represent the group mean and *shaded ribbons* the SEM. For each participant (eight epochs, 29 channels), power spectral density was calculated using Welch’s method and averaged first across channels for each epoch, then across epochs. These participant-level averages were then used to compute the final group statistics. Power is presented on a decibel scale (10 ⋅ log_10_ [μV^2^/Hz]).

At baseline, POD+ had, compared to POD–, lower beta band AECc (β = −0.36; 95% CI, −0.65 to −0.08; p_FDR_ = 0.04; fig. 2). This difference represented a medium effect size. While comparable effect sizes were observed for relative delta (β = −0.31; 95% CI, −0.60 to −0.02; p_FDR_ = 0.08) and theta power (β = 0.32; 95% CI, 0.04 to 0.59; p_FDR_ = 0.08), these differences were not statistically significant. See figure 3 for the group-averaged power spectral density plots at baseline and follow-up. Other qEEG characteristics showed no significant baseline differences between groups (Supplemental Digital Content 3 and 4, https://links.lww.com/ALN/E450). Exploratory electrode-level LMMs revealed that, in POD+ compared to POD–, the reduction in beta AECc was spatially concentrated over central and parietal regions (6 of 29 electrodes, p_FDR_ < 0.05) and increased theta power was concentrated over posterior and temporal regions (7 of 29 electrodes, p_FDR_ < 0.05) (Supplemental Digital Content 5, https://links.lww.com/ALN/E450).

### Effect of Surgery on qEEG Characteristics

No significant differences in qEEG characteristics were observed over time between surgical and nonsurgical patients, indicating that neither anesthesia nor surgery had any effects on the qEEG characteristics that were studied. No main effect of timepoint was found, and all time by group interaction effects were nonsignificant (*P*_unadjusted_ > 0.05), indicating that there were no significant changes over time, and that changes in qEEG characteristics over time did not differ significantly between surgical and nonsurgical patients (Supplemental Digital Content 3 and 4, https://links.lww.com/ALN/E450).

### Effect of POD on qEEG Characteristics

Analysis of qEEG characteristics revealed no statistically significant time by POD interactions for any of the qEEG measures (*p*_*unadjusted*_ > 0.05; Supplemental Digital Content 3, https://links.lww.com/ALN/E450). However, examination of effect sizes did reveal a medium effect for beta spectral variability (β = 0.31; 95% CI, 0.00 to 0.63; P_*unadjusted*_ = 0.05), suggesting a possible trend for greater increases in beta spectral variability over time in POD+ *versus* POD– .

## Discussion

In this prospective multicenter study, we analyzed preoperative EEG to identify qEEG markers of a propensity for POD and assessed whether POD or surgery induces persistent neurophysiological changes. First, preoperative resting-state EEG revealed that future POD+ patients showed statistically significant reductions in beta band functional connectivity measured using the AECc (β = −0.36, *P* = 0.04) compared to patients who did not develop POD. Nonsignificant medium effect sizes (β ≈ 0.3) were observed for reduced relative delta power and increased relative theta power. Second, we observed no significant long-term alterations in any of the measured qEEG characteristics at 3-month follow-up. This suggests that in our cohort, neither the occurrence of POD nor the surgical procedure itself resulted in lasting changes as assessed with resting-state EEG.

Our preoperative findings should be interpreted in the context of our study cohort, which consisted of a relatively healthy, cognitively intact, and functionally independent older-age population. At baseline, future POD+ patients were slightly older and had higher American Society of Anesthesiologists Physical Status scores, but no other major clinical or demographic differences distinguished them from the POD– group. In line with the minimal baseline clinical differences, the observed preoperative qEEG differences were also subtle, with modest effect sizes. These results align with the “disconnection syndrome” hypothesis, which posits that delirium arises when precipitants act upon preexisting vulnerability.^5,6,9,45^ We propose that the subtle nature of the baseline qEEG differences accurately reflects the low degree of preexisting vulnerability in this cohort. The development of delirium may have been driven less by severe baseline predisposition and more by precipitating factors, specifically the prolonged surgical duration.^46^ This interpretation is supported by findings from Ning *et al.*,^21^ who successfully predicted POD using preoperative qEEG metrics and cognitive scores in a cohort where POD+ patients had significantly greater baseline cognitive impairment compared to POD– patients. Greater cognitive impairment reflects higher delirium predisposition, which likely accounts for the more pronounced preoperative EEG differences in their cohort compared to our cognitively intact population.

The fact that we observed significant reduced preoperative beta AECc in future POD+ compared to POD– suggests that beta AECc may be sensitive to early, latent vulnerability. Previous studies have established beta AECc as a highly sensitive and reproducible marker for distinguishing Alzheimer’s dementia from healthy controls, reporting significantly reduced beta AECc in the dementia population.^47^ Given that cognitive impairment is a strong predisposing factor for delirium,^25^ the preoperative decrease we observed might reflect a similar latent network fragility. A recent study investigated reliability of several functional connectivity measures in resting-state EEG. They demonstrated that beta AECc exhibits moderate temporal reliability during a 6-week interval, supporting its utility as a stable baseline trait, whereas the stability across different resting-state EEG conditions during the same measurement was distinctively low.^48^ We postulate that difference does not merely reflect measurement noise but indicates that the beta AECc might be relatively sensitive to physiologic context. Because beta AECc is sensitive to shifts in resting-state conditions, it may be able to detect the subtle, neurophysiological signatures of delirium predisposition within this cohort. Interestingly, this preoperative state contrasts with the acute phase of delirium, where recent evidence indicates beta AECc is significantly increased.^11^ Although more research is needed, the contrast between preoperative decreased beta AECc and increased beta AECc during acute delirium suggests that this metric might be sensitive to the different neurophysiological phases of delirium.

The absence of qEEG changes 3 months postoperatively suggests that the pronounced EEG alterations typically seen during delirium are transient, at least in the current subset of patients. This finding aligns with previous work demonstrating the normalization of EEG after the acute episode. Kim *et al.*^19^ observed a decreased median frequency 5 days postoperatively that normalized after 1 month, and Nielsen *et al.*^49^ noted that delirium resolution was associated with the reappearance of beta activity and reduced delta activity in continuous EEG recordings. Our findings do contrast with studies that reported long-term EEG changes after delirium in nursing home residents.^22,23^ However, these discrepancies likely arise from key methodologic differences. The current study utilized 32-channel EEG in a relatively healthy surgical cohort, whereas these two previous studies used 2-channel EEG in older adults with probable preexisting cognitive decline.

Other neuroimaging techniques offer complementary insights into brain structure and functional activity after POD. The current study is part of a larger multicenter project that utilized multimodal neuroimaging, including EEG, fMRI, and structural MRI.^28^ Previous studies in this cohort have demonstrated gray matter loss^50^ and reduced resting-state fMRI connectivity strength but no differences in functional network efficiency and integration^24^ 3 months after delirium. The discrepancy with our EEG findings could reflect the brain’s capacity for functional compensation, where the brain preserves its electrophysiologic network integrity despite damage to the underlying gray matter and its associated metabolic support system, at least up to a certain threshold.^6^ Alternatively, our findings may indicate that the measures used lack the sensitivity to detect subtle changes against a background of natural functional connectivity fluctuations.^51^

A strength of this study is that it represents a large longitudinal multicenter study investigating a wide variety of global EEG characteristics in a surgical population. However, several limitations warrant consideration. First, despite the large sample size of the cohort, the number of POD+ patients was relatively small, which limited the statistical power to detect subtle differences. Second, we did not record EEG during acute POD episodes, limiting our ability to confirm whether the characteristic EEG alterations during delirium were present in our cohort. However, there is robust evidence for these patterns during (postoperative) delirium,^3,9^ including previous work from our research team.^7,8,10,17^ Third, the 3-month interval between surgery and follow-up EEG may have missed transient neurophysiological changes in the immediate postdelirium period, although this interval allowed us to assess whether any such changes persisted long-term. Future studies with multiple timepoints are needed to better characterize the recovery trajectory. Finally, it is important to note that our study was conducted in a cohort of relatively healthy and functionally independent older adults undergoing elective surgery. While our results are valid for this specific demographic group, they may not be generalizable to frailer patient populations with severe preexisting cognitive impairment, which typically face a higher baseline risk of delirium.

In conclusion, this study identifies reduced preoperative beta AECc as a subtle marker of neurophysiological vulnerability for POD, extending the disconnection syndrome hypothesis for delirium to preexisting network strength. Network disruptions assessed with EEG typically seen during acute delirium seem not to result in long-term neurophysiological changes, as no significant longitudinal changes or differential trajectories were observed between groups in this cohort of functionally independent older adults. Future studies are needed to explore the timeline and extent of persistent neurophysiological alterations, particularly in diverse delirium populations and across different timeframes.

### Research Support

The study was supported by the Biomarker Development for Postoperative Cognitive Impairment in the Elderly (BioCog) project, funded by the European Union's Seventh Framework Programme for Research and Technological Development (grant No. HEALTH-F2-2014-602461), and by ZonMw (the Netherlands Organisation for Health Research and Development, The Hague, the Netherlands; grant No. 09120012110032).

### Competing Interests

The authors declare no competing interests.

### Reproducible Science

Full protocol available at: j.vandera-2@umcutrecht.nl. Raw data available at: j.vandera-2@umcutrecht.nl.

## Supplemental Digital Content

Supplemental Digital Content, https://links.lww.com/ALN/E450

## Supplementary Material
