# Supplementary material for: Quantitative Electroencephalogram Changes before and after Postoperative Delirium: A Prospective, Multicenter Cohort Study
Source: Anesthesiology. 2026 Mar 9;144(6):1380–90. doi: 10.1097/ALN.0000000000006026 (PMC13155210; doi:10.1097/ALN.0000000000006026)
Supplement: Supplementary file 1 [file aln-144-1380-s001.pdf]

Supplementary material

Quantitative EEG changes preceding and following postoperative delirium: a prospective, multicenter, cohort study

Julia Van der A [1,2], Yorben Lodema [2], Lotus Ten Bosch [1,2], Anna B. Van Heesch [1,2], Simone J.T. Van Montfort [1], Ilse M.J. Kant [1,4], Claudia Spies [5], Edwin Van Dellen [2,3], Arjen J.C. Slooter[1,6]

1 - Department of Intensive Care Medicine, University Medical Center Utrecht Brain Center, University Medical Center Utrecht, Utrecht University, Utrecht, The Netherlands

2 - Department of Psychiatry and University Medical Center Utrecht Brain Center, University Medical Center Utrecht, Utrecht University, Utrecht, The Netherlands

3 - Department of Neurology and Vrije Universiteit Brussel, UZ Brussel, Brussel, Belgium.

4 - Department of Radiology and University Medical Center Utrecht Brain Center, University Medical Center Utrecht, Utrecht University, Utrecht, The Netherlands

5 - Department of Anesthesiology and Operative Intensive Care Medicine (CCM, CVK), Charité - Universitätsmedizin Berlin, Berlin, Germany.

6 - Department of Psychiatry, University Medical Center Groningen and University of Groningen, Groningen, The Netherlands

Supplementary Material 1. Electrode Groupings for Regions of Interest (ROIs)

**Region of interest (ROI)**	**Electrodes**
Frontal	Fp1, Fp2, F7, F3, Fz, F4, F8, FC1, FC2, FC5, FC6
Central	T7, C3, Cz, C4, T8, CP1, CP2, CP5, CP6
Parietal	P7, P3, Pz, P4, P8, POz
Occipital	O1, O2, Oz

Supplementary Material 2. Demographic characteristics of complete cases versus cases with missing data.

The following table summarizes demographic characteristics of patients who did not have missing data at baseline or follow-up, and those who did not complete both baseline and follow-up assessments or had only one EEG of sufficient quality, or had no timepoint of good EEG data (excluded cases).

	Complete cases (n = 166)	Missing cases (n = 164)	Excluded cases (n = 34)
Characteristics at baseline			
Center, Utrecht, n (%)	119 (72%)	41 (25%)	6 (18%)
Female, n (%)	58 (35%)	65 (40%)	10 (29%)
Age (years)	71 [68–75]	73 [69–75]	70 [68-74]
Diabetes mellitus, n (%)	23 (14%)	37 (23%)	8 (24%)
Body mass index	26 [23–28]	27 [24–29]	27 [25-29]
Transient ischemic attack or stroke, n (%)	19 (11%)	12 (7%)	3 (9%)
Charlson comorbidity index	0 [0–2]	1 [0–2]	2 [0-2]
Barthel index	100 [100–100]	100 [100–100]	100 [100-100]
Mini-Mental State Examination	29 [28-30]	29 [28–30]	29 [28-30]
Premorbid IQ[a]	107 [99–115]	104 [94–109]	100 [88-104]
Alcohol misuse, n (%)	9 (5%)	11 (7%)	1 (3%)
Geriatric Depression Scale	1 [0–2]	1 [0–3]	1 [0-2]
ASA physical status, n (%)			
1	17 (10%)	4 (2%)	1 (3%)
2	96 (58%)	108 (66%)	25 (74%)
3	53 (32%)	52 (32%)	5 (15%)
Surgery characteristics			
Surgical specialty, n (%)			
Cardiothoracic	33 (20%)	16 (10%)	1 (3%)
Intra-abdominal	58 (35%)	45 (27%)	11 (35%)
Orthopedic	47 (28%)	55 (34%)	6 (19%)
Other	28 (17%)	48 (29%)	13 (42%)
Duration of anesthesia (min)	237 [170–331]	150 [86–236]	149 [88-235]
Length of hospital stay (days)	5 [3–8]	6 [3–9]	6 [3-8]
Length of ICU stay (days)	0 [0–0]	0 [0–0]	0 [0-0]
Delirious, n (%)	26 (16%)	33 (20%)	2 (7%) ±
Characteristics at follow-up			
Mortality before follow-up, n (%)	-	8 (5%)	0 (0%)

Supplementary table 1. Values are median [25th–75th percentile] unless stated otherwise. [a] Estimated using the Dutch version of the National Adult Reading Test; Complete cases+ = participants completed baseline and follow-up assessment. Missing cases = participants who did not complete both baseline and follow-up assessments. IQ = intelligent quotient. ± = data was missing for 5 participants.

Supplementary Material 3. Linear mixed models: standardized beta coefficients (β) and 95% confidence interval (CI) for all fixed factors

	Centre	Age	Sex	Timepoint	Delirium	Operated	Timepoint × Group (Controls)	Timepoint × Group (POD+)
**Peak frequency**	0.04 [-0.17, 0.24]	-0.22 [-0.32, -0.12]*	-0.14 [-0.34, 0.06]	-0.03 [-0.11, 0.06]	-0.16 [-0.44, 0.13]	0.21 [-0.51, 0.08]	0.03 [-0.16, 0.22]	-0.06 [-0.29, 0.16]
Relative power								
Delta	-0.21 [-0.41, 0.00]	-0.005 [-0.11, 0.10]	0.17 [-0.03, 0.38]	-0.01 [-0.10, 0.07]	-0.31 [-0.60, -0.02]*	-0.04 [-0.26, 0.34]	-0.08 [-0.26, 0.11]	-0.12 [-0.33, 0.09]
Theta	0.04 [-0.16, 0.24]	0.17 [0.07, 0.26]*	0.22 [0.02, 0.41]*	0.04 [-0.03, 0.12]	0.32 [0.04, 0.59]*	0.02 [-0.27, 0.31]	0.02 [-0.14, 0.19]	0.12 [-0.07, 0.31]
Alpha	-0.07 [-0.28, 0.13]	-0.10 [-0.20, 0.00]	-0.12 [-0.33, 0.09]	-0.02 [-0.10, 0.05]	0.09 [-0.20, 0.38]	-0.02 [-0.32, 0 .28]	0.09 [-0.08, 0.26]	0.10 [-0.09, 0.30]
Beta	0.45 [0.24, 0.65]*	-0.02 [-0.12, 0.08]	-0.35 [-0.56, -0.15]*	0.01 [-0.06, 0.09]	-0.08 [-0.36, 0.21]	-0.05 [-0.35, 0.24]	-0.09 [-0.25, 0.07]	-0.18 [-0.36, 0.01]
PLI								
Delta	-0.46 [-0.64, -0.28]*	0.02 [-0.06, 0.11]	0.50 [0.33, 0.67]*	-0.11 [-0.28, 0.05]	-0.22 [-0.50, 0.07]	-0.03 [-0.31, 0.26]	-0.23 [-0.61, 0.15]	0.000 [-0.41, 0.41]
Theta	0.02 [-0.18, 0.22]	0.01 [-0.08, 0 .11]	0.26 [0.07, 0.46]*	-0.02 [-0.11, 0.08]	0.04 [-0.24, 0.33]	0.003 [-0.29, 0.28]	0.07 [-0.15, 0.28]	0.10 [-0.15, 0.34]
Alpha	-0.09 [-0.30, 0.11]	-0.06 [-0.16, 0.04]	0.11 [-0.09, 0.31]	-0.08 [-0.17, 0.02]	-0.04 [-0.33, 0.25]	0.01 [-0.29, 0.31]	0.14 [-0.07, 0.35]	0.17 [-0.07, 0.41]
Beta	-0.20 [-0.38, -0.01]*	-0.11 [-0.20, -0.02]*	0.12 [-0.07, 0.30]	-0.005 [-0.17, 0.16]	-0.03 [-0.33, 0.27]	0.44 [0.14, 0.74]*	-0.21 [-0.59, 0.17]	0.13 [-0.29, 0.55]
AECc								
Delta	0.16 [-0.03, 0.35]	0.09 [0.00, 0.18]	-0.22 [-0.40, -0.04]*	-0.01 [-0.18, 0.15]	0.003 [-0.30, 0.29]	-0.36 [-0.65, -0.06]*	0.07 [-0.44, 0.31]	-0.03 [-0.44, 0.39]
Theta	0.11 [-0.08, 0.31]	0.02 [-0.08, 0.11]	-0.25 [-0.45, -0.06]*	-0.04 [-0.17, 0.08]	0.12 [-0.17, 0.40]	0.02 [-0.27, 0.31]	0.14 [-0.15, 0.42]	-0.09 [-0.41, 0.23]
Alpha	0.08 [-0.11, 0.28]	-0.14 [-0.24, -0.05]*	-0.23 [-0.43, -0.04]*	-0.02 [-0.14, 0.10]	-0.18 [-0.47, 0.10]	0.06 [-0.23, 0.35]	-0.18 [-0.45, 0.09]	-0.14 [-0.45, 0.17]
Beta	-0.24 [-0.44, -0.05]*	-0.21 [-0.30, -0.11]*	-0.26 [-0.45, -0.06]*	-0.02 [-0.13, 0.09]	-0.36 [-0.65, -0.08]*	-0.10 [-0.39, 0.19]	0.06 [-0.18, 0.30]	-0.11 [-0.38, 0.17]
Spectral variability								

	Centre	Age	Sex	Timepoint	Delirium	Operated	Timepoint × Group (Controls)	Timepoint × Group (POD+)
Delta	0.09 [-0.12, 0.29]	-0.04 [-0.13, 0.06]	-0.11 [-0.32, 0.09]	-0.06 [-0.16, 0.04]	0.27 [-0.02, 0.56]	-0.10 [-0.39, 0.20]	0.19 [-0.04, 0.41]	0.11 [-0.14, 0.37]
Theta	-0.13 [-0.33, 0.06]	-0.18 [-0.27, -0.08]*	-0.11 [-0.30, 0.09]	-0.10 [-0.23, 0.02]	-0.19 [-0.48, 0.10]	-0.14 [-0.43, 0.16]	0.21 [-0.08, 0.49]	-0.07 [-0.39, 0.25]
Alpha	-0.12 [-0.33, 0.09]	-0.03 [-0.13, 0.07]	0.15 [-0.06, 0.36]	0.01 [-0.08, 0.10]	-0.16 [-0.46, 0.14]	0.08 [-0.23, 0.38]	-0.10 [-0.30, 0.10]	-0.09 [-0.32, 0.14]
Beta	-0.49 [-0.69, -0.29]*	-0.07 [-0.16, 0.03]	0.31 [0.12, 0.51]*	-0.09 [-0.21, 0.04]	-0.08 [-0.37, 0.21]	-0.09 [-0.39, 0.20]	0.21 [-0.07, 0.48]	0.31 [0.00, 0.63]
**Approximate entropy**	0.48 [0.28, 0.69]*	-0.06 [-0.16, 0.04]	-0.46 [-0.66, -0.26]*	0.02 [-0.06, 0.11]	0.11 [-0.18, 0.40]	-0.07 [-0.37, 0.22]	-0.008 [-0.20, 0.18]	-0.07 [-0.29, 0.15]

Supplementary Table 2. Values represent post hoc standardized beta coefficients (β) with 95% confidence intervals from linear mixed models. Each model included the qEEG characteristic as the dependent variable, with fixed effects for center (Utrecht = reference, Berlin), age (years), sex (female = reference, male), anesthesia duration (minutes), timepoint (T1 [preoperative/baseline] = reference, T3 [postoperative follow-up]) and Group (POD− = reference, POD+, Controls). Participant was included as a random effect (random intercept model), with interaction terms for timepoint × group. Models were first fit using raw values, and standardized β coefficients were then derived using the posthoc method in the effectsize package in R (version 0.8.9), expressing effects in standard deviation units.

* significant at $p \leq 0.05$.

Supplementary Material 4. Topographical plots demonstrating the spatial distribution of quantitative EEG measures

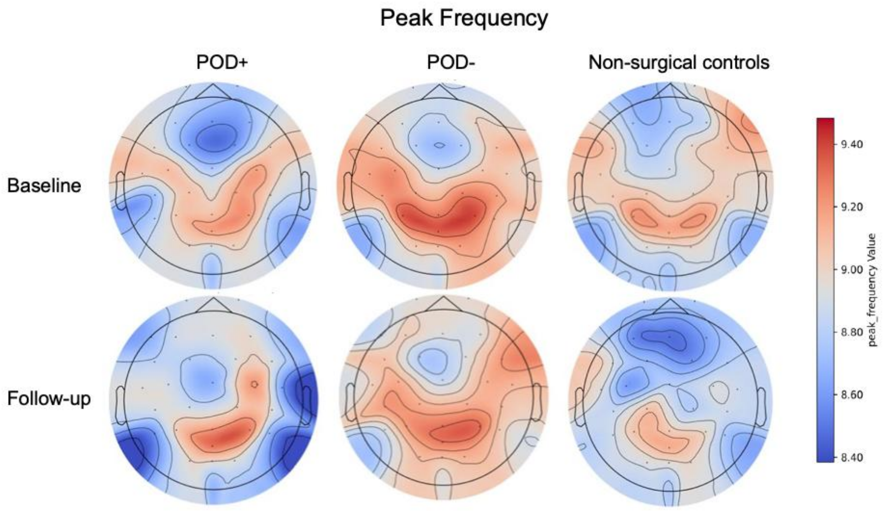

Figure 1. The heat maps display the median peak frequency across three groups: patients with postoperative delirium (POD+), patients without POD (POD-), and non-surgical controls at two different timepoints (baseline/preoperative and follow-up/three months postoperative). None of the groups showed a significant change over time.

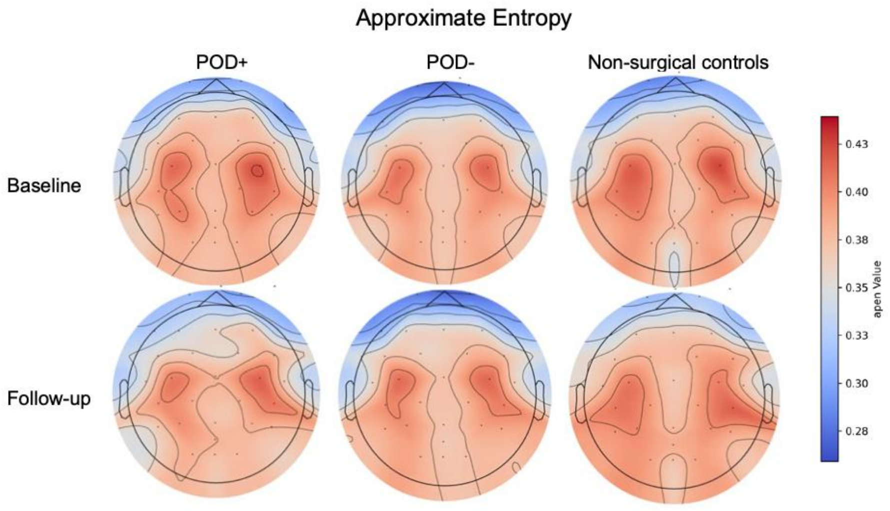

Figure 2. The heat maps display the median approximate entropy across three groups: patients with postoperative delirium (POD+), patients without POD (POD-), and non-surgical controls at two different timepoints (baseline/preoperative and follow-up/three months postoperative). None of the groups showed a significant change over time.

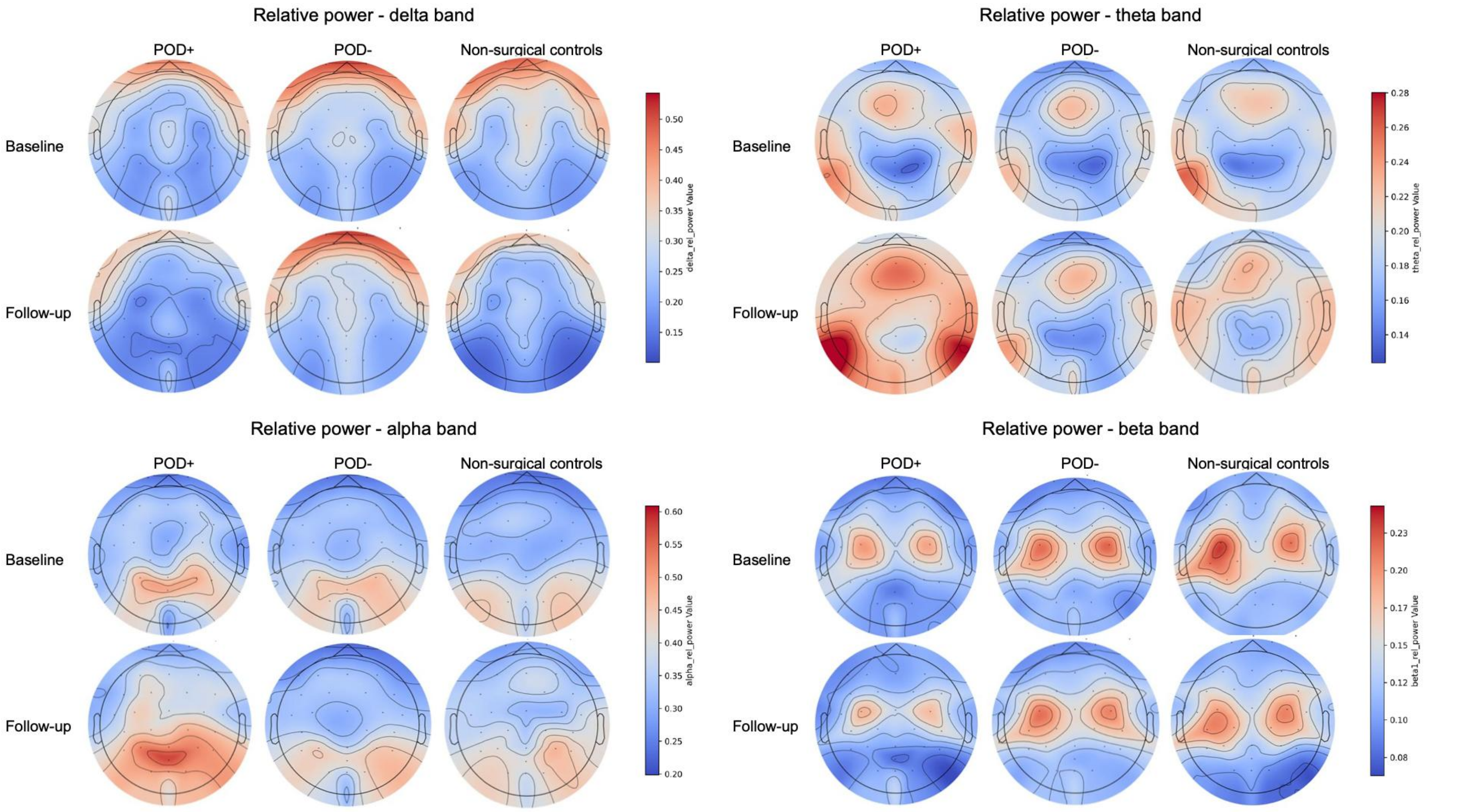

Figure 3. The heat maps display the median relative power for the delta (0,5-4 Hz, δ), theta (4-8 Hz, θ), alpha (8-13 Hz, α), and beta (13-20 Hz, β) frequency bands across three groups: patients with postoperative delirium (POD+), patients without POD (POD-), and non-surgical controls at two different timepoints (baseline/preoperative and follow-up/three months postoperative). Color scales for the heat maps were standardized per frequency band.

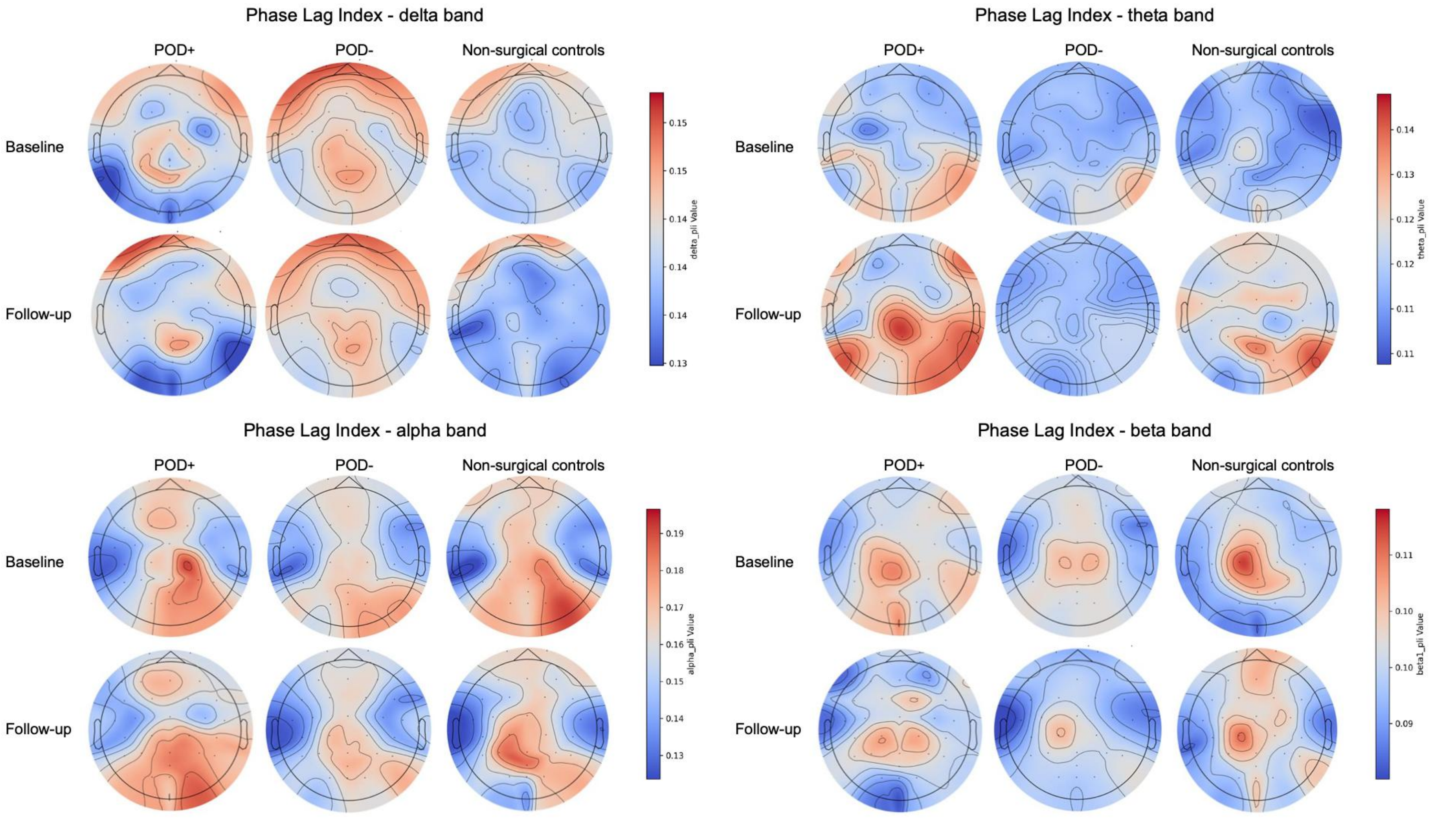

Figure 4. The heat maps display the median phase lag index (PLI) for the delta (0.5-4 Hz, δ), theta (4-8 Hz, θ), alpha (8-13 Hz, α), and beta (13-20 Hz, β) frequency bands across three groups: patients with postoperative delirium (POD+), patients without POD (POD-), and non-surgical controls at two different timepoints (baseline/preoperative and follow-up/three months postoperative). Color scales for the heat maps were standardized per frequency band.

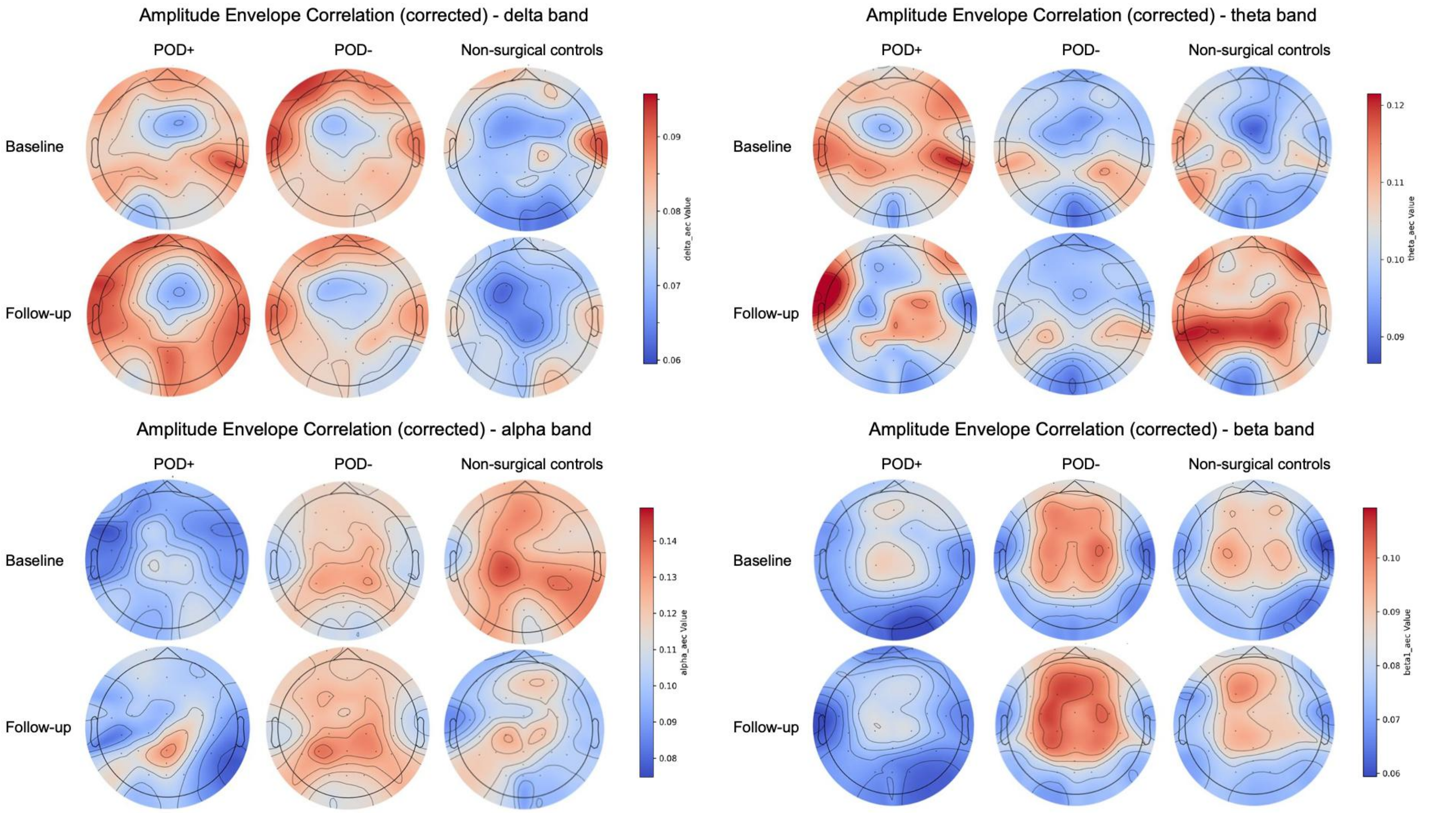

Figure 5. The heat maps display the median amplitude envelope correlation (corrected) for the delta (0.5-4 Hz, δ), theta (4-8 Hz, θ), alpha (8-13 Hz, α), and beta (13-20 Hz, β) frequency bands across three groups: patients with postoperative delirium (POD+), patients without POD (POD-), and non-surgical controls at two different timepoints (baseline and follow-up). Color scales for the heat maps were standardized per frequency band. The beta band AECc was also shown in the main paper.

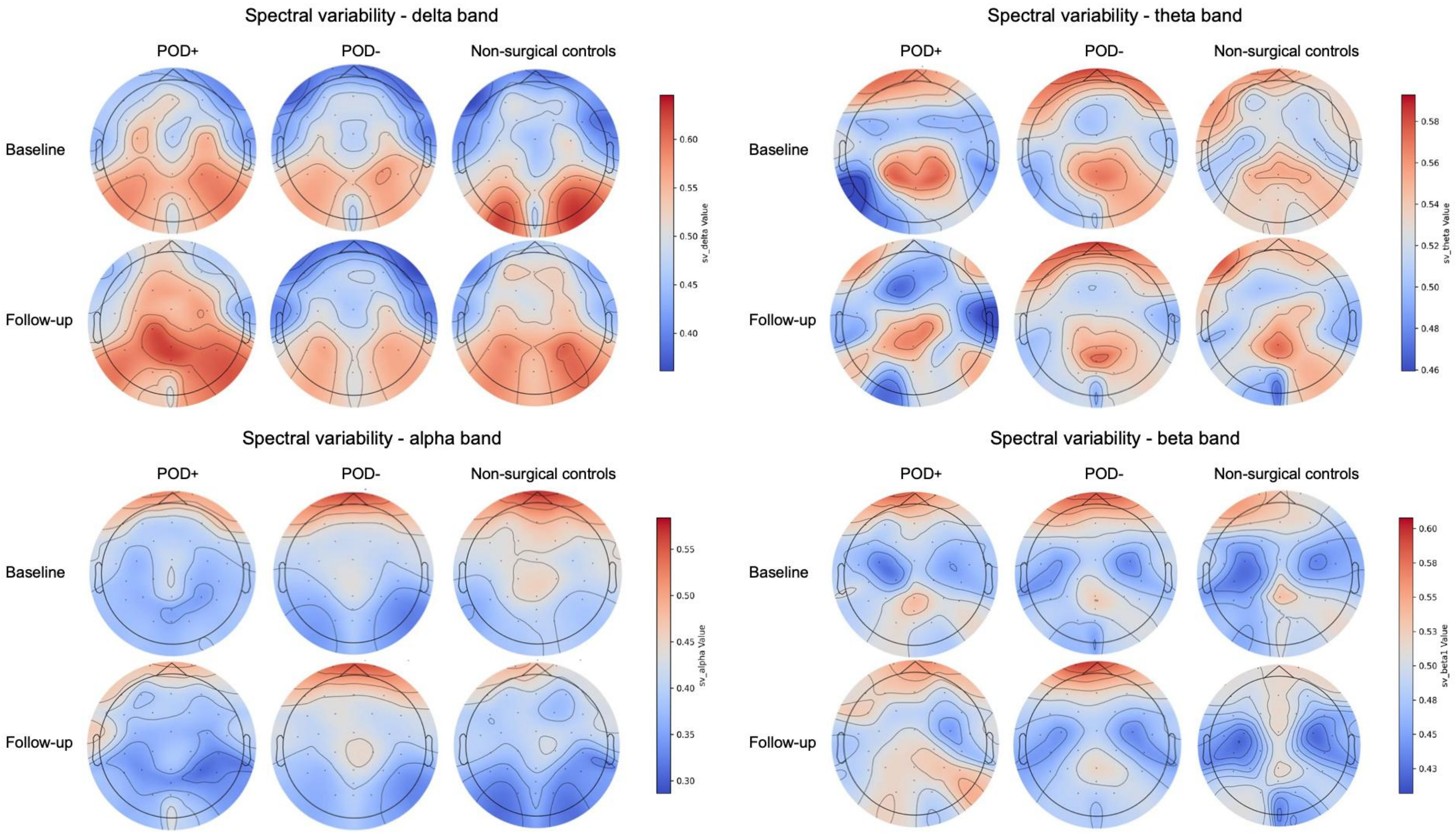

Figure 6. The heat maps display the median spectral variability for the delta (0.5-4 Hz, δ), theta (4-8 Hz, θ), alpha (8-13 Hz, α), and beta (13-20 Hz, β) frequency bands across three groups: patients with postoperative delirium (POD+), patients without POD (POD-), and non-surgical controls at two different timepoints (baseline and follow-up). Color scales for the heat maps were standardized per frequency band.

Supplementary Material 5. Electrode-level LMM t-statistic maps for preoperative POD+ vs. POD- group differences

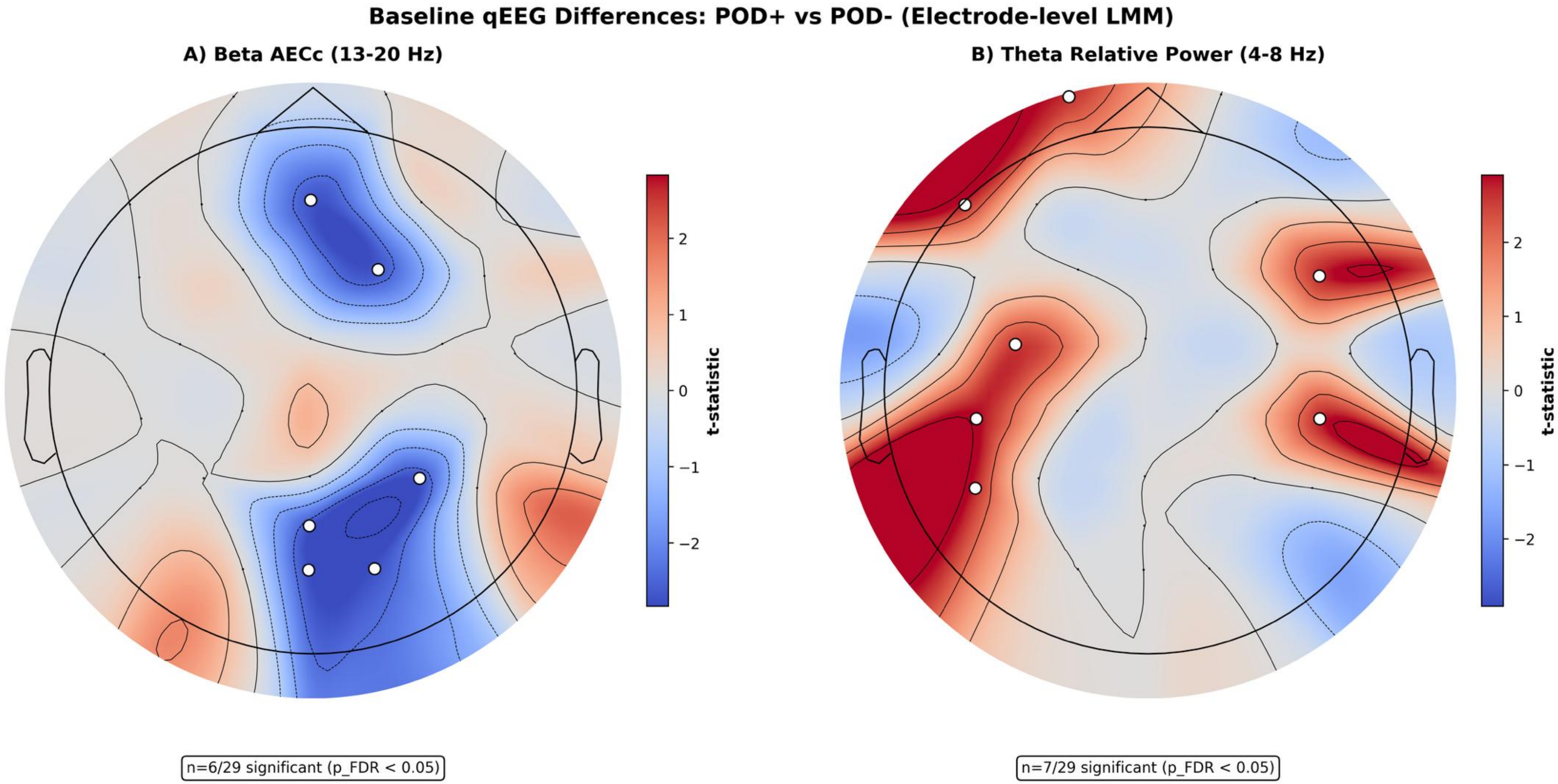

Supplementary Material 5. A) Beta corrected Amplitude Envelope Correlation (AECc) (13–20 Hz) at baseline (preoperative) B) Theta relative power (4–8 Hz) at baseline (preoperative). Color reflects the t-statistic from the LMM group term (patients who will later experience postoperative delirium, POD+, vs. patients who will not develop postoperative delirium, POD-), with blue indicating lower values in POD+ and red indicating higher values. White dots indicate electrodes that showed significant differences after correction for multiple comparisons across electrodes, which was done using the False Discovery Rate (pFDR < 0.05).
